# Effect of Different Luting Agents on the Retention of Lithium Disilicate Ceramic Crowns

**DOI:** 10.3390/ma8041604

**Published:** 2015-04-07

**Authors:** Nicola Mobilio, Alberto Fasiol, Francesco Mollica, Santo Catapano

**Affiliations:** 1 Dental Clinic, University of Ferrara, c.so Giovecca 203, 44121 Ferrara, Italy; E-Mails: albertofasiol@yahoo.it (A.F.); santo.catapano@unife.it (S.C.); 2 Department of Engineering, University of Ferrara, v. Saragat 1, 44122 Ferrara, Italy; E-Mail: francesco.mollica@unife.it

**Keywords:** crowns, dental cement, lithium disilicate crowns, all-ceramics

## Abstract

No studies are available that evaluate the retention of disilicate crowns according to different cementation procedures. The purpose of this study was to measure the retention of lithium disilicate crowns cemented using two different cementation systems. Twenty extracted mandibular premolars were prepared. Anatomic crowns were waxed and hot pressed using lithium disilicate ceramic. Teeth were divided into two groups (*n* = 10): (1) self-curing luting composite and (2) glass-ionomer cement (GIC). After cementation, the crowns were embedded in acrylic resin block with a screw base. Each specimen was pulled along the path of insertion in Universal Testing Machine. Failure load in Newtons (N) and failure mode were recorded for each specimen. Failure mode was classified as decementation or fracture. Failure load data were analyzed using one-way analysis of variance (ANOVA). Failure modes were compared using Pearson’s Chi-square test. Mean failure load was 306.6(±193.8) N for composite group and 94.7(±48.2) N for GIC group (*p* = 0.004). Disilicate crown cemented with luting composite most often failed by fracture; otherwise, crown cemented with glass-ionomer cement most often failed by decementation (*p* = 0.02). Disilicate full crown cemented with luting composite showed higher failure load compared with conventional cementation with glass-ionomer cement.

## 1. Introduction

Lithium disilicate is an all-ceramic material that combines good mechanical properties and excellent aesthetic results. This ceramic is indicated for single crowns, veneers and inlays, and compared with other glass-ceramics, it demonstrates very good performance [1]. This material may be pressed or milled, and it may be produced as a monolithic restoration or veneered for a highly esthetic outcome.

*In vitro* studies evaluated the fracture resistance of disilicate crowns. Monolithic lithium disilicate crowns showed very good results in comparison to both zirconia and metal-ceramic crowns [2,3], and they are considered a reliable treatment alternative even for a posterior, high load-bearing area [4].

From a clinical perspective, disilicate single crowns are commonly used both in the anterior and posterior region [5,6]. A recent study found that anterior and posterior lithium disilicate restorations, with a mean follow-up of 3 years, showed successful rates ranged from 95.39% to 100% [7]. A review concluded that disilicate single crowns showed excellent short-term survival rates, even if the evidence for medium-term survival is limited [8].

It is generally assumed that the clinical success of these all-ceramic restorations is affected by the type of cementation [9,10]. A strong advantage for using disilicate is adhesive cementation. As a glass-ceramic, the inner surface of the lithium disilicate may be etched using fluoridric acid to increase the surface energy and consequently, the bond strength [11,12]. Nevertheless, traditional luting by glass-ionomer cement is also indicated for disilicate, and medium-term survival rates respective to the type of cementation are not conclusive [6].

Although many studies have investigated shear bond strength of disilicate specimens [13,14], no studies are currently available evaluating the retention of disilicate crowns according to different cementation procedures. The aim of this study was to measure the retention of lithium disilicate ceramic crowns cemented onto extracted human teeth using two different cementation systems.

## 2. Results and Discussion

The mean (and standard deviation) of failure loads are shown in Table 1. There was a significant difference between composite and glass-ionomer cement (GIC) group (*F* (1, 18) = 11.26, *p* = 0.004).

**Figure 1:**
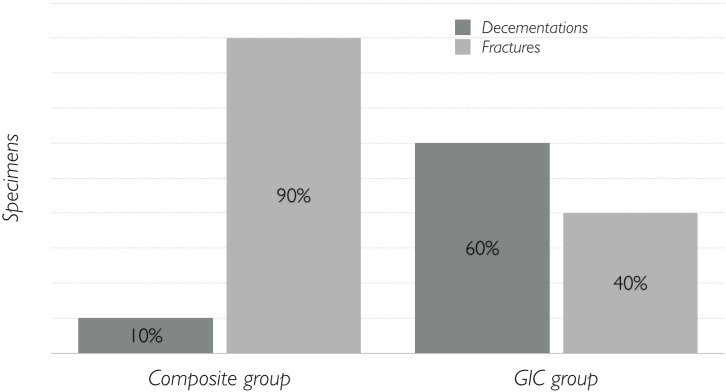
Results (percentages) divided according to failure mode.

Failure modes are shown in Figure 1. Disilicate crown cemented with luting composite most often failed by fracture, otherwise crown cemented with glass-ionomer cement most often failed by decementation (*p* = 0.02). For all the fractured specimens, the fracture occurred apical to the margin of the crown, quite horizontally. Among decemented specimens, most of them in GIC group showed an adhesive failure, with the cement left in the inner surface of the crown, and only two specimens showed a cohesive failure, with the cement left both on the tooth and crown. The single decemented specimen in the composite group showed the same adhesive failure.

**Table 1:** Mean failure loads (standard deviation) in N.

Groups	Mean (sd)
Composite group	306.6 (193.8)
GIC group	94.7 (48.2)

Traditionally, definitive cementation is considered a fundamental step among restorative procedures. The correct cement selection and correct cementation procedure are crucial for adequate marginal sealing and proper retention of the restoration in time [15].

In evaluating the retentive capacity of disilicate crown cemented on natural teeth, the primary challenge is related to how to hold the crown. Previous studies have evaluated the retentive capacity of metal or zirconia crowns: these crowns were designed to include a ring or a peduncle for grabbing [16,17]. Such a solution cannot be applied for all ceramic crowns because the thinnest part of the ceramic would break during the test. This problem was resolved by designing a more convex crown in order for the acrylic resin to go beyond the undercut and provide uniform traction all around the ceramic. The present study is the first one to evaluate the retentive capacity of all ceramic crowns in such a manner.

The results of the present study showed a higher failure load for disilicate crowns cemented with luting composite compared with a more conventional cementation procedure using a glass-ionomer cement. No previous studies have evaluated the retention of disilicate crowns cemented on natural teeth, and thus a comparison may be difficult to perform. In a study evaluating the retention of zirconia crowns cemented on natural teeth, luting composite showed a higher retentive strength compared with glass-ionomer cements [16].

An interesting aspect of the presented results is the type of failure. The expected outcome was the decementation of the crown from the tooth, *i.e.*, the fracture of the cement layer. In such a scenario, it would be logical to calculate the surface area of each abutment to extrapolate the debonding force per mm^2^ (MPa). Even if this approach was largely reported in literature [16,17,18], it presents many computational and practical problems because the surfaces of the teeth are complex, non-planar, resulting in a mix of tensile and shear forces. Furthermore, decementation occurred in only one specimen in composite group, while it was the predominant failure mode in GIC group. Such a difference in failure mode was found to be statistically significant. In this context, it would not make any sense to compare the debonding force of a surface that actually did not debond but fractured. For this reason, it was decided to compare failure loads of both groups. The present finding may actually suggest a completely different biomechanical behavior between the two luting procedures: the ceramic crown etched and cemented by luting composite cement become part of the tooth. Thus, the failure load of a crown cemented in that fashion may be equal to or even higher than the inner tensile strength of a natural tooth.

Some limits of the present study need to be discussed. Only two cements were tested. Even if the GIC may be considered quite representative of its category, this cannot be accepted for luting composite. Indeed, many luting composites exist that are very different in composition and characteristics, so using different cements could lead to different results. The convergence angle between the axial walls of tooth preparation was standardized to 10 degrees. It may be expected that a more pronounced taper angle, where bond strength is the most important factor, may produce different results. Despite the careful selection of teeth and the use of standard protocols according to the manufacturing instructions, our results showed high standard deviation. This was most likely due to intrinsic differences present in natural teeth. Nevertheless, other studies on natural teeth have shown similar results [16]. Another aspect is the cement layer: the present results are valid for the specific thickness of cement, which was standardized for all specimens, but a different thickness of layer may result in different results. Finally, it is possible that after thermocycling, different results may be expected due to the potential degradation of the cement interface. Thus, further studies are necessary to investigate these possibilities.

## 3. Experimental Section

Twenty single-rooted mandibular premolars, extracted for periodontal or orthodontic reasons, were collected after excluding teeth with caries and/or previous restorations. After removing dental plaque, calculus, and periodontal tissues with ultrasonic instruments and curettes, the teeth were stored in physiological solution until further use.

The root of each tooth was embedded in a self-curing acrylic resin block (ProBase Cold, Ivoclar Vivadent AG, Schaan, Liechtenstein) up to 2 mm below the cement-enamel junction (CEJ) and with its long axis perpendicular to the base of the block.

Preparation of the teeth was standardized using a diamond bur mounted onto a surveyor. Each tooth was prepared for a 1.25 mm circumferential chamfer and a total occlusal convergence angle of 10 degrees between axial walls. The finishing line was at the CEJ level.

An anatomic crown was waxed on each tooth and then hot pressed using lithium disilicate ceramic (IPS e.max PRESS, Ivoclar Vivadent AG, Schaan, Liechtenstein, batch number S06095). The spacer was applied according to the manufacturer’s instructions.

The specimens were randomly divided into two groups (*n* = 10) according to different cementation systems:

Composite group: self-curing luting composite (Multilink Automix, Ivoclar Vivadent AG).
GIC group: glass-ionomer cement (Vivaglass Cem, Ivoclar Vivadent AG).

The cementation procedures strictly followed the manufacturing instructions. In composite group, the inner surface of the ceramic crowns were etched with 5% hydrofluoridric acid (IPS Ceramic gel, Ivoclar Vivadent AG) for 20 s and then rinsed and cleaned in pure alcohol in an ultrasonic bath for ten minutes. Thus, the crowns were treated with a universal primer (Monobond Plus, Ivoclar Vivadent AG) for 60 s and then dried with hot air. The teeth were cleaned and dried, and then adhesive (Multilink Primer, Ivoclar Vivadent AG) was brushed on and dried. The cement (Multilink Automix, Ivoclar Vivadent AG) was applied onto the inner surface of the crowns and then seated onto the prepared teeth. Each crown was subjected to an axial seating force of 50 N using an Instron 4467 Universal Testing Machine (ITW Test and Measurement Italia S.r.l., Torino, Italy) until the end of polymerization, according to manufacturing.

In GIC group, the glass-ionomer cement (Vivaglass Cem, Ivoclar Vivadent AG) was mixed and applied onto the internal surface of each crown and then seated onto the teeth. Each crown was subjected to an axial seating force of 50 N using an Instron 4467 Universal Testing Machine until the end of setting according to manufacturing.

**Figure 2:**
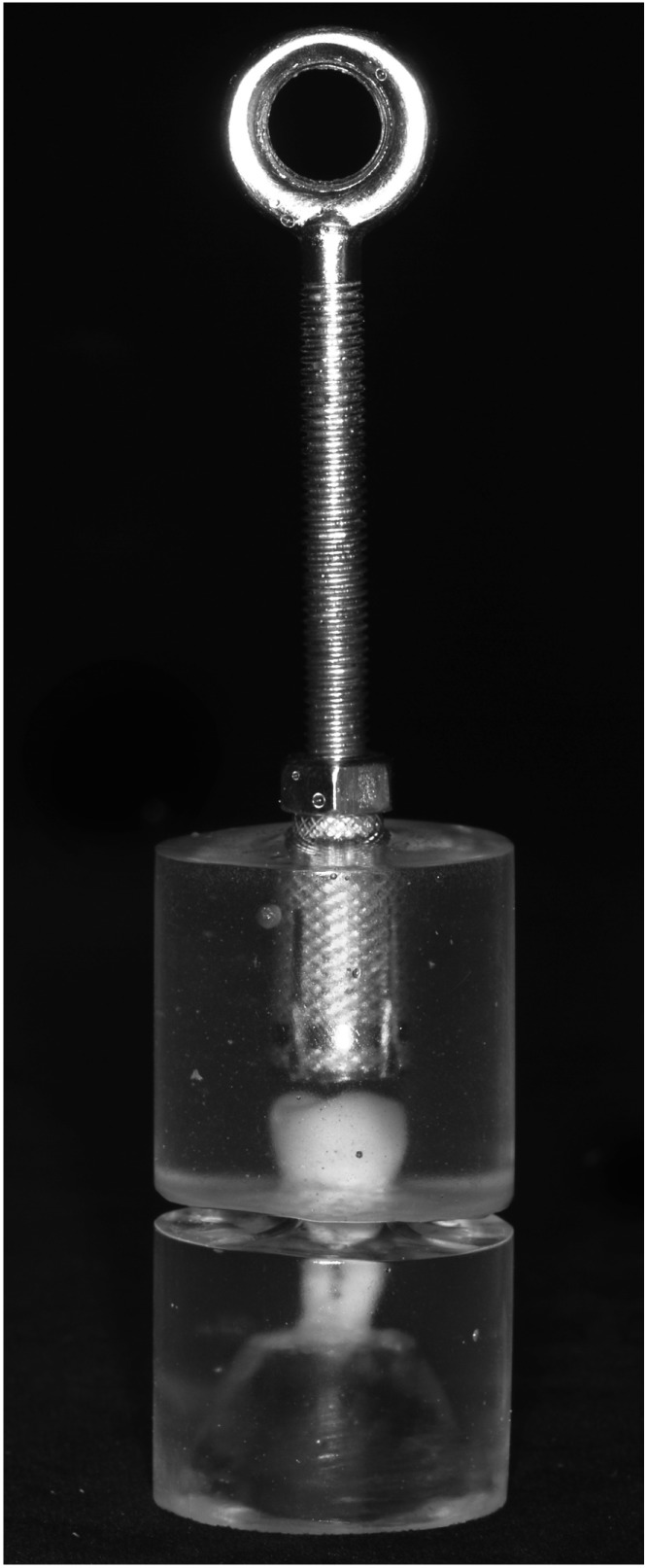
Specimen ready to be tested.

After cementation, the crowns were stored in water at 37 °C for one week. Then they were embedded in an autopolymerizing acrylic resin block (ProBase Cold, Ivoclar Vivadent AG). At the top of this block, a screw base was incorporated with its long axis parallel to the long axis of the tooth (Figure 2).

**Figure 3:**
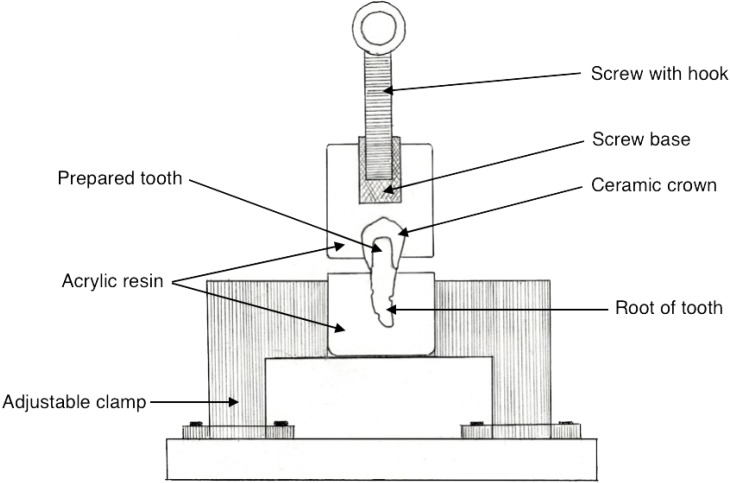
Schematic drawing of the experimental apparatus.

**Figure 4:**
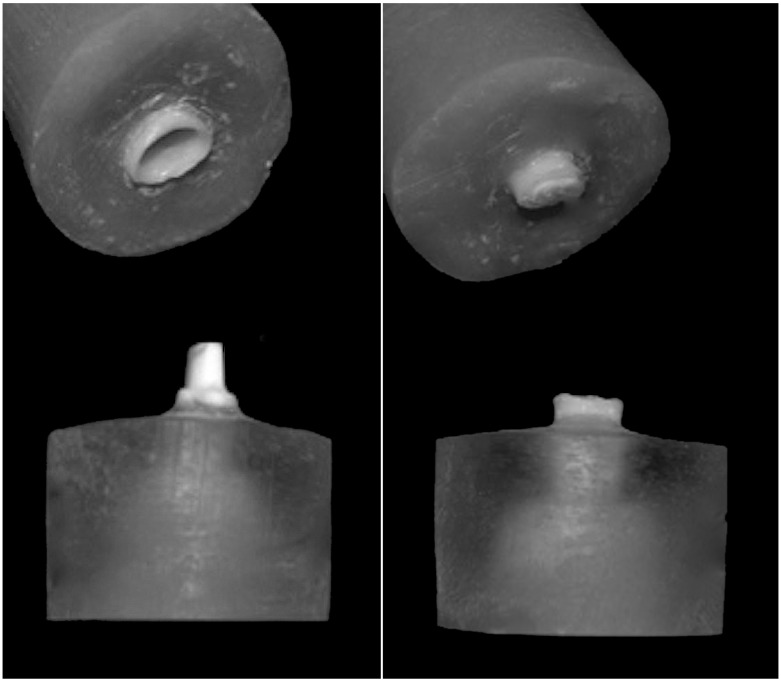
Examples of different failure modes: decementation (**left**) and fracture (**right**).

The retention was investigated using an Instron 4467 Universal Testing Machine. Each specimen was positioned in a metal holder with the long axis of the tooth parallel to the load direction (Figure 3). Each upper block was pulled along the path of insertion of the crown with a crosshead speed of 1 mm/min. Failure load in Newtons (N) and failure mode were recorded for each specimen. Failure mode was classified as decementation or fracture (Figure 4).

Data were statistically analyzed using the IBM SPSS software v.22 for Mac OSX (IBM, Armonk, NY, USA). The Kolmogorov-Smirnov test was used to confirm the normality of data distribution. Failure load data were analyzed with one-way analysis of variance (ANOVA). Failure modes were compared using Pearson’s Chi-square test. The level of significance was set at 0.05.

## 4. Conclusions

Within the limits of this *in vitro* study, lithium disilicate full crowns cemented with luting composite showed higher failure loads compared with conventional cementation with glass-ionomer cement. Furthermore, disilicate crown cemented with luting composite most often failed by fracture, and crown cemented with glass-ionomer cement most often failed by decementation.

## References

[1] Denry I., Holloway J.A. (2010). Ceramics for dental applications: A review. Materials.

[2] Zahran M., El-Mowafy O., Tam L., Watson P.A. (2008). Fracture strength and fatigue resistance of all-ceramic molar crowns manufactured with CAD/CAM technology. J. Prosthodont..

[3] Guess P.C., Zavanelli R.A., Silva N.R., Bonfante E.A., Coelho P.G., Thompson V.P. (2010). Monolithic CAD/CAM lithium disilicate *versus* veneered Y-TZP crowns: Comparison of failure modes and reliability after fatigue. Int. J. Prosthodont..

[4] Schultheis S., Strub J.R., Gerds T.A., Guess P.C. (2013). Monolithic and bi-layer CAD/CAM lithium-disilicate *versus* metal-ceramic fixed dental prostheses: Comparison of fracture loads and failure modes after fatigue. Clin. Oral Investig..

[5] Valenti M., Valenti A. (2009). Retrospective survival analysis of 261 lithium disilicate crowns in a private general practice. Quintessence Int..

[6] Gehrt M., Wolfart S., Rafai N., Reich S., Edelhoff D. (2012). Clinical results of lithium-disilicate crowns after up to 9 years of service. Clin. Oral Investig..

[7] Fabbri G., Zarone F., Dellificorelli G., Cannistraro G., de Lorenzi M., Mosca A. (2014). Clinical evaluation of 860 anterior and posterior lithium disilicate restorations: Retrospective study with a mean follow-up of 3 years and a maximum observational period of 6 years. Int. J. Periodont. Restor. Dent..

[8] Pieger S., Salman A., Bidra A.S. (2014). Clinical outcomes of lithium disilicate single crowns and partial fixed dental prostheses: A systematic review. J. Prosthet. Dent..

[9] Blatz M.B., Sadan A., Kern M. (2003). Resin-ceramic bonding: A review of the literature. J. Prosthet. Dent..

[10] Blatz M.B., Oppes S., Chiche G., Holst S., Sadan A. (2008). Influence of cementation technique on fracture strength and leakage of alumina all-ceramic crowns after cyclic loading. Quintessence Int..

[11] Ozcan M., Vallittu P.K. (2003). Effect of surface conditioning methods on the bond strength of luting cement to ceramics. Dent. Mater..

[12] Tian T., Tsoi J.K., Matinlinna J.P., Burrow M.F. (2014). Aspects of bonding between resin luting cements and glass ceramic materials. Dent. Mater..

[13] Nagai T., Kawamoto Y., Kakehashi Y., Matsumura H. (2005). Adhesive bonding of a lithium disilicate ceramic material with resin-based luting agents. J. Oral Rehabil..

[14] Cekic-Nagas I., Canay S., Sahin E. (2010). Bonding of resin core materials to lithium disilicate ceramics: The effect of resin cement film thickness. Int. J. Prosthodont..

[15] Rosenstiel S.F., Land M.F., Fujimoto J. (2006). Contemporary Fixed Prosthodontics.

[16] Ernst C.P., Cohnen U., Stender E., Willershausen B. (2005). *In vitro* retentive strength of zirconium oxide ceramic crowns using different luting agents. J. Prosthet. Dent..

[17] Ali A.O., Kelly J.R., Zandparsa R. (2012). The influence of different convergence angles and resin cements on the retention of zirconia copings. J. Prosthodont..

[18] Dahl B.L., Oilo G. (1986). Retentive properties of luting cements: An *in vitro* investigation. Dent. Mater..

